# Harnessing digital health to achieve equitable and efficient health systems

**DOI:** 10.2471/BLT.24.293051

**Published:** 2025-02-01

**Authors:** Viroj Tangcharoensathien, Alain Labrique, Divya Lakhotia, Rapeepong Suphanchaimat, Walaiporn Patcharanarumol

**Affiliations:** a International Health Policy Program, Ministry of Public Health, Tiwanon Road, Nonthaburi, 11000, Thailand.; b Department of Digital Health and Innovation, World Health Organization, Geneva, Switzerland.

Progress towards achieving the sustainable development goals (SDGs) is off track, with only five years remaining until 2030.^1^ Bold actions are needed for countries to achieve the SDG targets. One promising avenue is the application of digital technologies in the implementation of the SDGs, which could directly support more than two thirds of the targets.^2^

Digital health technologies can redefine and re-engineer the tools needed to create a better future for all; they can, for example, drive earlier diagnoses and interventions, improve outcomes, and support and engage patients.^3^ This theme issue of the *Bulletin of the World Health Organization* explores how digitalization shapes health systems, highlighting opportunities for innovation while addressing the challenges of inequity.

Health determinants are no longer static; they have evolved alongside the digital ecosystems, interacting in ways that are reshaping individual and population health. A scoping review in this issue proposes a conceptual framework that captures this complexity, placing health at its core and illustrating how traditional determinants – social, political, economic and commercial – blend with emerging digital determinants and together influence health outcomes and equity. For policy-makers, this framework is a tool to navigate these changes, offering pathways to mitigate harms and provide opportunities to enhance health equity.^4^ Policy-makers must also proactively respond to the emergence of generative artificial intelligence to harness its benefits while mitigating its risks, such as job displacement, which negatively affect health.^5^

The transformative potential of digital health innovations is most evident in how they overcome service delivery challenges in low-resource settings. In Guangzhou, China, a mobile health application has improved access to voluntary human immunodeficiency virus testing and counselling. The success of the application lies in its thoughtful design, informed by the needs of both providers and users, and its integration into existing health systems.^6^

India’s Scan and Share initiative shows another successful lesson from the field. By leveraging mobile technology and quick response codes, the initiative reduced outpatient waiting times from hours to minutes across nearly 17 000 health-care facilities across 35 states and territories.^7^ Digital solutions, grounded in simplicity and scalability, can transform health-care delivery at a national level.

A perspective introduces digital health diplomacy to minimize digital health gaps, emphasizing the need for multilateral cross-border collaborations for data sharing, capacity-building and strengthening digital public infrastructure in underserved regions.^8^ Such efforts enable innovations such as Aga Khan University’s telemedicine model, which connects critical care doctors with rural and remote health-care teams in Afghanistan, Kenya, Pakistan and United Republic of Tanzania. Using basic technologies such as instant messaging technologies and videotelephony, the model provided over 6000 consultations, improving survival rates for critically ill patients.^9^

Despite these successes, the adoption of digital health technologies by health workers, particularly in low- and middle-income countries, remains uneven. A systematic review highlights both facilitators and barriers to adoption – with trust, incentives and robust infrastructure playing critical positive roles. Conversely, concerns about usability, performance and lack of self-efficacy hinder uptake. Addressing these gaps requires investment in training, organizational support and participatory and inclusive design.^10^

To scale digital health responsibly, an article suggests that low- and middle-income countries need to focus on three key enablers. First, governance needs to prioritize local autonomy and accountability. Second, infrastructure must be agile and adaptable. Finally, human security must remain the guiding principle, ensuring that digitalization enhances health equity rather than exacerbating disparities.^11^

The challenge of this era is not simply about adopting digital technologies but doing so in a way that aligns with the principles of equity, inclusivity and sustainability. A systematic review highlights the effectiveness and cost–effectiveness of digital health interventions for managing rheumatic diseases, demonstrating their potential to improve disease control, patient adherence and self-efficacy. However, the review also underscores a stark digital divide: while high-income countries employ advanced tools like telemedicine platforms, low- and middle-income countries rely on simpler, more accessible technologies like mobile messaging, reflecting infrastructure and resource gaps.^12^ As explained in the perspective on inclusive digital health, each decision – whether designing a platform, implementing a policy or fostering a partnership – must aim towards a healthier, more equitable future.^13^
